# Alveolar cytokines and interferon autoantibodies in COVID-19 ARDS

**DOI:** 10.3389/fimmu.2024.1353012

**Published:** 2024-03-20

**Authors:** Trine B. Jonassen, Sofie E. Jørgensen, Nikki H. Mitchell, Trine H. Mogensen, Ronan M. G. Berg, Andreas Ronit, Ronni R. Plovsing

**Affiliations:** ^1^ Department of Anesthesiology and Intensive Care, Copenhagen University Hospital-Amager and Hvidovre Hospitals, Hvidovre, Denmark; ^2^ Department of Infectious Diseases, Aarhus University Hospital (AUH), Aarhus, Denmark; ^3^ Department of Biomedicine, Aarhus University, Aarhus, Denmark; ^4^ Department of Clinical Biochemistry, Copenhagen University Hospital-Amager and Hvidovre, Hvidovre, Denmark; ^5^ Department of Biomedical Sciences, Faculty of Health and Medical Sciences, University of Copenhagen, Copenhagen, Denmark; ^6^ Department of Clinical Physiology and Nuclear Medicine, Copenhagen University Hospital-Rigshospitalet, Copenhagen, Denmark; ^7^ Centre for Physical Activity Research, Copenhagen University Hospital-Rigshospitalet, Copenhagen, Denmark; ^8^ Neurovascular Research Laboratory, Faculty of Life Sciences and Education, University of South Wales, Pontypridd, United Kingdom; ^9^ Department of Infectious Diseases, Copenhagen University Hospital-Amager and Hvidovre Hospitals, Hvidovre, Denmark; ^10^ Department of Clinical Medicine, Faculty of Health and Medical Sciences, University of Copenhagen, Copenhagen, Denmark

**Keywords:** bronchoalveolar lavage fluid, coronavirus disease 2019, interferons, inflammation, acute respiratory distress syndrome, autoantibodies

## Abstract

**Background:**

Type I interferon (IFN-I) and IFN autoantibodies play a crucial role in controlling SARS-CoV-2 infection. The levels of these mediators have only rarely been studied in the alveolar compartment in patients with COVID-19 acute respiratory distress syndrome (CARDS) but have not been compared across different ARDS etiologies, and the potential effect of dexamethasone (DXM) on these mediators is not known.

**Methods:**

We assessed the integrity of the alveolo-capillary membrane, interleukins, type I, II, and III IFNs, and IFN autoantibodies by studying the epithelial lining fluid (ELF) volumes, alveolar concentration of protein, and ELF-corrected concentrations of cytokines in two patient subgroups and controls.

**Results:**

A total of 16 patients with CARDS (four without and 12 with DXM treatment), eight with non-CARDS, and 15 healthy controls were included. The highest ELF volumes and protein levels were observed in CARDS. Systemic and ELF-corrected alveolar concentrations of interleukin (IL)-6 appeared to be particularly low in patients with CARDS receiving DXM, whereas alveolar levels of IL-8 were high regardless of DXM treatment. Alveolar levels of IFNs were similar between CARDS and non-CARDS patients, and IFNα and IFNω autoantibody levels were higher in patients with CARDS and non-CARDS than in healthy controls.

**Conclusions:**

Patients with CARDS exhibited greater alveolo-capillary barrier disruption with compartmentalization of IL-8, regardless of DXM treatment, whereas systemic and alveolar levels of IL-6 were lower in the DXM-treated subgroup. IFN-I autoantibodies were higher in the BALF of CARDS patients, independent of DXM, whereas IFN autoantibodies in plasma were similar to those in controls.

## 1 Introduction

The pathophysiological changes accompanying acute respiratory distress syndrome (ARDS), a common heterogeneous cause of respiratory failure with high mortality, have been of interest to clinical researchers for decades (1). A hallmark of ARDS is increased permeability of protein-rich fluid and cells across the lung endothelium to the alveoli, including injury-inducing neutrophils (1).

In viral respiratory infections, immune-mediated factors may contribute to disease control, and are associated with disease severity (2). During the COVID-19 pandemic, the pathophysiologic role of biomarkers and potential therapeutic options to alter these biomarkers in COVID-19 ARDS (CARDS) (3–5) have become a growing area of research, with a focus on systemic and alveolar pro-inflammatory mediators, such as interleukin (IL)-6, IL-8, and tumor necrosis factor (TNF)-α.

Some studies have also compared the profiles of inflammatory mediators in patients with CARDS with non-COVID-19 ARDS (non-CARDS) (6). However, few studies have focused on measuring alveolar levels of interferons (IFNs) and autoantibodies against IFN. Interferons are crucial for viral control due to their ability to suppress viral replication and facilitate the activation of dendritic cells while also enhancing the functions of lymphocytes and macrophages (7, 8). The levels of these mediators have only occasionally been studied at alveolar levels in CARDS patients (9); however, to our knowledge, they have not been compared across different ARDS etiologies or according to whether dexamethasone (DXM) was used as an intervention.

To further understand the integrity of the alveoli-capillary membrane in CARDS and non-CARDS patients, we studied the extent of protein-rich alveolar edema and assessed whether systemically administered DXM has an overall impact on membrane integrity in CARDS. Moreover, we investigated the differences in alveolar epithelial lining fluid (ELF) concentrations of cytokines, including type I, II, III, IFNs, and IFN autoantibodies.

## 2 Methods

### 2.1 Study population, design, and ethics

Three groups were included in the study (CARDS, non-CARDS, and healthy controls). In all three groups, standardized bronchoalveolar lavage (BAL) and blood sampling were performed at a given time point. Patients with CARDS were included in the intensive care unit (ICU) of Hvidovre Hospital, Denmark, during the first and second COVID-19 waves. The inclusion criteria were age >18 years, polymerase chain reaction-confirmed SARS-CoV-2 infection, moderate-to-severe ARDS (Berlin definition 2012 (10)), and less than 72 h of invasive mechanical ventilation. CARDS patients were further divided into two groups depending on whether they were treated with DXM, as the study enrollment covered the period before and after DXM was implemented as a standard of care. Non-CARDS patients included intubated ICU patients aged >18 years with either ARDS (Berlin definition 2012 (10)) and/or sepsis (Sepsis-2 criteria (11)) and less than 48 h of mechanical ventilation. Further details of the patient and healthy control groups, which included healthy male subjects, have been previously described (12). Oral and written informed consent was obtained from next-of-kin (CARDS, non-CARDS) or healthy controls prior to participation and approval was given by the Ethical Committee of the Capital Region of Copenhagen (H-2-2009-131; H-2-2011-021; H-2-0023-159).

### 2.2 Bronchoalveolar lavage procedure and sampling

A standardized BAL was performed in a subsegment of the right middle lobe (CARDS and non-CARDS patients) or lingula (healthy controls) by an experienced proceduralist, as previously described (12). In all groups, 150 ml of isotonic saline was instilled for each BAL procedure, aspirated immediately, pooled into a sterile container on ice, and processed within 15 min of collection.

### 2.3 Epithelial lining fluid and cytokine concentrations

Urea may be used for determining the amount of ELF, i.e., the thin layer of fluid covering the mucosa of the alveoli and the small and large bronchioles, because the concentration of urea in plasma equals the concentration in ELF under steady state conditions (13, 14). Because lavaged BALF is a mixture of instilled saline and ELF, the *in situ* concentration of urea in ELF is greater than the measured concentration in BALF. To determine the *in situ* concentrations of mediators, the volume of a solute-specific concentration was calculated using the following method:


$$\text{Volume ELF}=\;\frac{\left[\text{Urea BALF}\right]\cdot \text{Volume BALF}}{\left[\text{Urea Plasma}\right]}$$

$$\left[\text{Solute ELF}\right]=\;\frac{\left[\text{Solute BALF}\right]\cdot \text{Volume BALF}}{\text{Volume ELF}}$$

where [Urea BALF] is the concentration of urea in BALF (mmol/L), Volume BALF is the lavaged return volume (mL), [Urea Plasma] is the concentration of urea in plasma (mmol/L), and [Solute BALF] is the solute concentration (e.g., TNF-α) in BALF (mmol/L).

### 2.4 Measurements of total protein, albumin, and urea in BALF

Total protein, albumin, and urea levels in the BALF samples were measured at the Department of Clinical Biochemistry, University Hospital Hvidovre, Denmark. All parameters were measured using the Cobas 8000 system (Roche Diagnostics GmbH, Germany). Total protein and albumin were analyzed using cerebrospinal fluid, and urea was analyzed using plasma. Frozen (−80°C) BALF-samples were thawed at the laboratory and analyzed immediately after, albeit some of the samples had to be re-centrifuged before analysis. Albumin concentration was reported as mg/L, total protein as g/L, and urea as mmol/L.

### 2.5 Cytokine detection

Cytokine levels of TNF-α, IL-1β, IL-6, IL-8, IFN-α2a, IFN-β, IFN-γ, and IFN-λ1 were measured in BALF and plasma, according to the manufacturer’s instructions (mesoscale U-PLEX assay, Meso Scale Diagnostics, USA). Notably, IFN levels (IFN-α2a, IFN-β, IFN-γ, and IFN-λ1) were not measured in healthy controls. In patients with CARDS, the potential live SARS-CoV-2 in BALF was inactivated by mixing BALF and 0.4% Triton-X-100 1:1 and incubating for 30 min at room temperature before analysis. Inactivated BALF samples were analyzed undiluted, and plasma was analyzed undiluted and 10× diluted.

### 2.6 IFN autoantibodies

To measure IFN autoantibodies in BALF and plasma, ELISA plates were coated with 1 µg/mL IFNα (130-093-874, Miltenyi Biotec) or IFNω (BMS304, Invitrogen) overnight at 4°C, followed by blocking with 5% skimmed milk. Plasma samples were diluted 50× in HPE buffer (M1940, Sanquin) before incubation. Bound autoantibodies were detected with HRP-conjugated goat anti-human IgG, IgA, IgM (GAHu/Ig/Fc/PO, Nordic-MUbio), and HRP substrate, SureBlue KPL (5120-0077, Sera care). BAL samples from patients with CARDS were diluted 3× in 0.4% Triton-X-100 and left for 30 min to ensure inactivation of potential SARS-CoV-2 present in the samples before incubation on the plates. A cut-off of 0.5 (blank-corrected OD450-OD630) was used to determine positivity in plasma samples based on data from previous studies (15). The cutoff value for determining positivity in BAL samples is unknown.

### 2.7 Statistics

Unless otherwise stated, continuous variables were expressed as median (interquartile range, IQR) and compared using the non-parametric Mann–Whitney test. Normality of distribution was not assumed, given the relatively small sample size. Accordingly, comparisons between groups were made using the non-parametric Kruskal–Wallis test, followed by Dunn’s test with Bonferroni correction for multiple comparisons between groups if a significant difference was found in the former. For statistical analysis, undetectable concentrations of cytokines measured in plasma or BALF were assigned an arbitrary value equivalent to 50% of the lower limit of detection. A p-value<0.05 was considered to represent a statistically significant difference. All analyses were performed using the R software version 4.2.1.

## 3 Results

### 3.1 Baseline characteristics

The baseline characteristics of patients and healthy controls are shown in **Table 1**. The median durations of invasive mechanical ventilation before the BAL procedure in patients with CARDS and non-CARDS were 26 [17–45] and 28 [18–39] h, respectively. There were no differences in age and sex between the two patient groups, but CARDS patients exhibited a larger impairment of gas exchange than to non-CARDS patients (PaO_2_/F_I_O_2_ 110 mmHg [88–149] vs. 162 mmHg [115–183], p = 0.03; **Table 1**). Furthermore, leucocyte and CRP levels were higher in both patient groups, with the highest levels observed in non-CARDS patients. Tocilizumab, a potent IL-6 inhibitor, was administered to three patients with CARDS, but neither inclusion nor omittance of these patients in the statistical analyses resulted in any change in leukocytes and CRP or concentration of cytokines in both plasma and BAL (data not shown). The baseline characteristics did not differ between the CARDS subgroups, except for a lower PaO_2_/F_I_O_2_ (P/F) ratio observed in the subgroup not receiving DXM (75 mmHg [63–82] vs. 125 mmHg [110–151], p<0.01).

**Table 1:** Baseline characteristics.

	CARDS n = 16	Non-CARDS n = 8	Healthy controls n = 15	P-value*
Age (years)	66 [56–72]	65 [62–68]	23 [22–23]	0.69
Sex, males (%)	75	63	100	0.56
Height (cm)	175 [171–179]	173 [167–180]	187 [182–189]	0.88
Weight (kg)	80 [72–87]	81 [68–93]	80 [73–86]	0.83
CRP (mg/L)	97 [56–125]	183 [129–266]	1 [1–1]	0.03
Leukocytes (10^9^/L)	11 [8–14]	24 [13–32]	5 [5–6]	0.03
Neutrophils (10^9^/L)	10 [8–12]	19 [11–26]	3 [2–3]	0.08
PaO_2_/F_I_O_2_ (mmHg)	110 [88–149]	162 [115–183]	–	0.03
SAPS II/SAPS III**	59 [56–69]	46 [38–66]	–	–
Mechanical ventilation before BAL (hours)	26 [17–45]	28 [18–39]	–	0.54
Symptoms before BAL (days)	12 [10–14]	–	–	–
DXM	12	–	–	–
DXM before BAL (days)	5 [3–5]	–	–	–
Tocilizumab	3	–	–	–

Baseline characteristics of critically ill patients with CARDS and non-CARDS and healthy controls. Notably, the CARDS patients (with or without DXM) did not differ with regard to baseline characteristics, except for the P/F-ratio, which was lower in the group not receiving DXM. Data are expressed as the median [IQR]. CRP, leukocytes and neutrophils were measured in the plasma at the time of the BAL procedure. DXM and tocilizumab were administered systemically according to national guidelines at the time of inclusion (DXM: 7.2 mg, IV bolus for a total of 10 days; tocilizumab: 8 mg/kg in 100 ml saline, IV infusion, single dose). *P-values refer to differences between critically ill patients with CARDS and non-CARDS. **SAPS III; CARDS patients, SAPS II; non-CARDS patients. BAL, bronchoalveolar lavage; CARDS, COVID-19 associated acute respiratory distress syndrome; CRP, C-reactive protein; DXM, dexamethasone; non-CARDS, non-COVID-19 associated acute respiratory distress syndrome and/or sepsis.

### 3.2 Alveolo-capillary membrane integrity—ELF volumes

**Figure 1:**
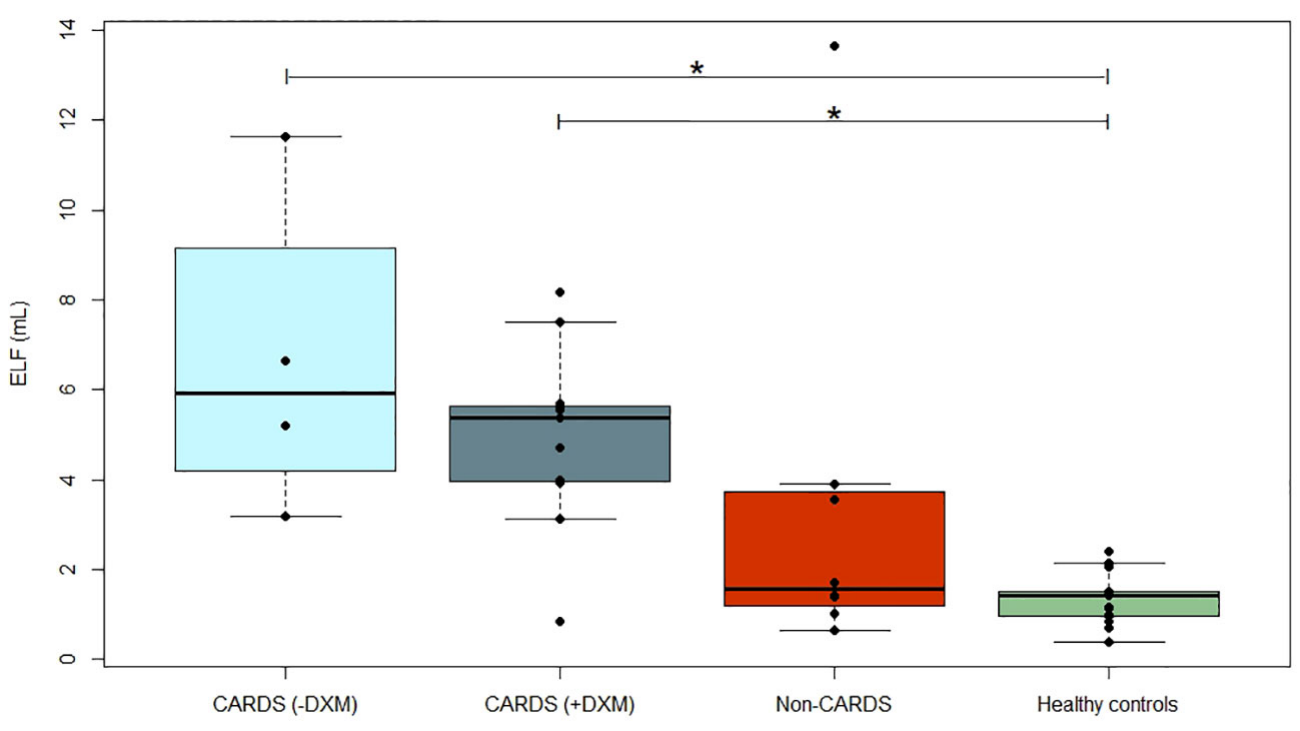
Epithelial lining fluid. Volumes (mL) of ELF in CARDS patients without (−DXM, n = 4) or with DXM treatment (+DXM, n = 12), non-CARDS critically ill patients (n = 8) and healthy controls (n = 15). *P<0.05 (Dunn’s test). Box plots visualize the median, first, and third quartiles (hinges), and whiskers extends from the hinge to the largest value no further than 1.5 ∗ IQR from the hinge, with data beyond plotted individually as outlying points. BAL, bronchoalveolar lavage; CARDS, COVID-19 associated acute respiratory distress syndrome; DXM, dexamethasone; ELF, epithelial lining fluid; non-CARDS, non-COVID-19 associated acute respiratory distress syndrome and/or sepsis.

The ELF volumes are shown in **Figure 1**. Higher volumes of ELF were found in both CARDS subgroups compared to healthy controls and appeared most pronounced in CARDS patients not receiving DXM, where a 5-fold higher value was observed (6.66 mL vs. 1.35 mL). A significant difference was found between the healthy controls and patients with CARDS without (p = 0.014) and with DXM treatment (p = 0.002). No differences were found between CARDS and non-CARDS patients, regardless of CARDS subgroup. Similarly, no differences were observed between patients with non-CARDS and healthy volunteers.

### 3.3 Alveolo-capillary membrane integrity—protein concentrations

**Figure 2:**
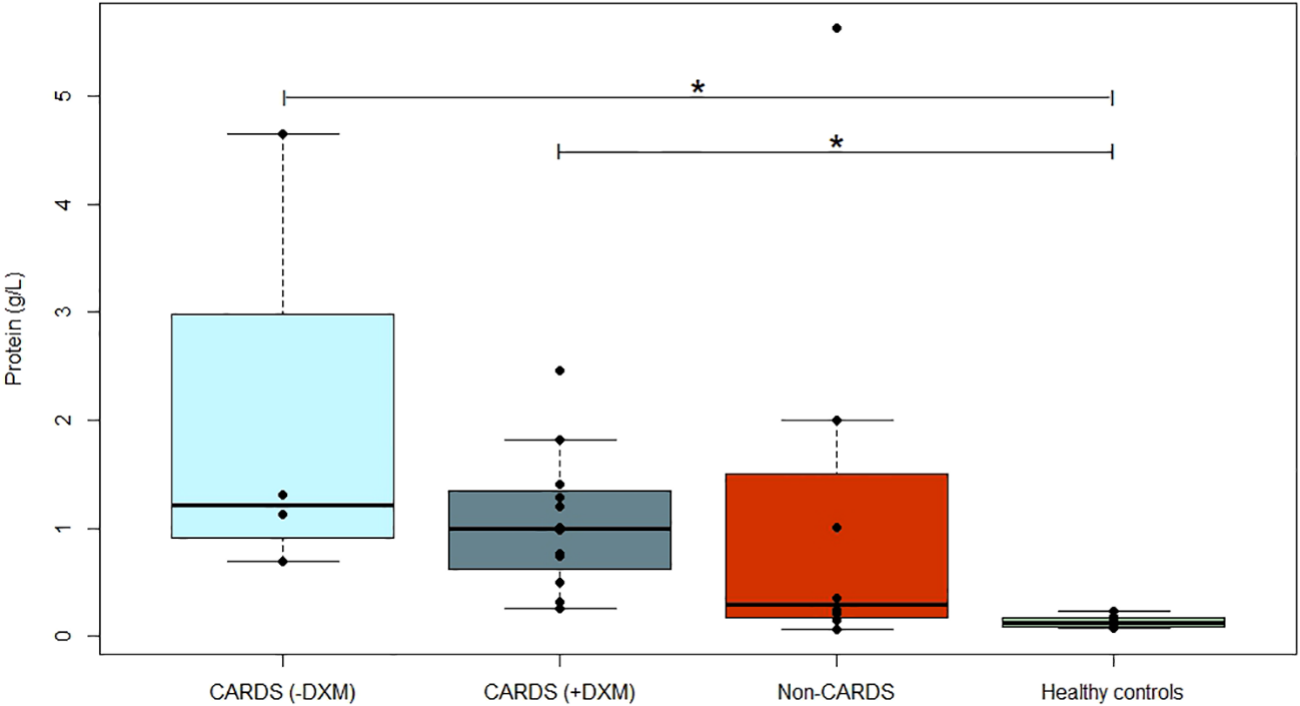
Alveolar concentrations of proteins. Concentration of alveolar protein (g/L) in CARDS patients without (−DXM, n = 4) or with DXM treatment (+DXM, n = 12), non-CARDS critically ill patients (n = 8), and healthy controls (n = 15). *P<0.05 (Dunn’s test). Box plots visualize the median, first, and third quartiles (hinges), and whiskers extend from the hinge to the largest value no further than 1.5 ∗ IQR from the hinge, with data beyond plotted individually as outlying points. ARDS, acute respiratory distress syndrome; BAL, bronchoalveolar lavage; CARDS, COVID-19 associated acute respiratory distress syndrome; DXM, dexamethasone; Non-CARDS, non-COVID-19 associated acute respiratory distress syndrome and/or sepsis.

The alveolar protein concentration was higher in CARDS patients than in healthy controls (−DXM: p = 0.004, +DXM: p<0.001), and a similar trend was observed for non-CARDS (p = 0.08), but there was no difference between the two patient groups (**Figure 2**).

Within the subgroup of CARDS patients, those who did not receive DXM tended to have higher protein concentrations (+23% compared to CARDS +DXM), although this was not statistically significant (p = 0.65). Albumin accounted for approximately 45% of the total protein in both subgroups.

### 3.4 Alveolar and systemic levels of interleukins

There were no differences in ELF-corrected alveolar concentrations of TNF-α and IL-1β between CARDS, non-CARDS, and healthy controls. Even though the median ELF-corrected alveolar IL-6 was higher in CARDS −DXM and lower in CARDS +DXM than in healthy volunteers, this was not statistically significant (p = 0.08; **Table 2**). However, the ELF-corrected concentration of IL-8 was higher in both CARDS and non-CARDS patients than in healthy controls but did not differ between the CARDS and non-CARDS subgroups (**Table 2**).

**Table 2:** Alveolar and plasma concentrations of cytokines and autoantibodies.

	*CARDS (−DXM)* *n = 4*	*CARDS (+DXM)* *n = 12*	*Non-CARDS* *n = 8*	*Healthy controls* *n = 15*	*P-value* *(Kruskal–Wallis)*
Interleukins (pg/mL) - BALF					
*TNF-α*	6 [4–18]	9 [3–18]	8 [2–20]	21 [14–28]	0.16
*IL-1β*	85 [44–178]	42 [25–51]	58 [3–356]	17 [10–28]	0.4
*IL-6*	5,701 [3,956–7,027]	726 [349–2,971]	2,464 [1,189–3,467]	1,186 [455–3,582]	0.08
*IL-8*	19,940 [13,116–25,052]*	16,100 [8,009–34,735]*	9,571 [3,838–55,123]*	1,461 [1,062–1,994]	<0.01
Interleukins (pg/mL)—Plasma					
*TNF-α*	1.9 [1.5–2.2]	0.9 [0.8–1.2]*	1.3 [0.7–2.5]	1.9 [1.67–2]	<0.01
*IL-1β*	0.4 [0.3–0.4]*	0.2 [0.1–0.4]*	0.2 [0.1–0.5]*	0.02 [0.02–0.04]	<0.001
*IL-6*	678 [15–1,398]*	11 [6–43]*	74 [33–1,142]*	0.98 [0.67–1.92]	<0.001
*IL-8*	27 [20–40]*	17 [12–24]*	88 [40–164]*	3.1 [2.6–3.6]	<0.001
Interferons (pg/mL)—BALF					
*IFN-λ1*	68 [7–158]	76 [60–159]	32 [13–49]		0.17
*IFN-α2a*	9 [5–13]	3 [2–3]	6 [2–10]		0.43
*IFN-β*	16 [12–23]	20 [11–25]	56 [16–136]		0.62
*IFN-γ*	9 [5–12]	19 [9–26]	73 [36–94]		0.11
Interferons (pg/mL)–Plasma					
*IFN-λ1*	4 [4–11]	11 [6–16]	2 [1–3]**		<0.01
*IFN-α2a*	0.2 [0.2–0.4]	0.3 [0.2–0.5]	0.2 [0.1–0.2]		0.13
*IFN-β*	5 [4–6]	7 [6–14]	4 [3–21]		0.61
*IFN-γ*	82 [59–95]	12 [3–19]	6 [2–17]		0.1
Autoantibodies (OD450–630 nm)—BALF					
*IFN-α*	0.20 [0.14–0.26]	0.29 [0.15–0.34]*	0.12 [0.12–0.26]	0.12 [0.11–0.15]	0.04
*IFN-ω*	0.1 [0.06–0.14]	0.15 [0.06–0.37]*	0.07 [0.03–0.09]	0.03 [0.03–0.04]	<0.01
Autoantibodies (OD450–630 nm)—Plasma					
*IFN-α*	0.02 [0.01–0.08]	0.09 [0.06–0.12]	0.05 [0.03–0.06*	0.13 [0.08–0.17]	<0.01
*IFN-ω*	0.06 [0.05–0.1]*	0.12 [0.1–0.22]	0.08 [0.06–0.08]*	0.16 [0.11–0.26]	<0.01

Interleukins (TNF-α, IL-1β, IL-6, and IL-8), interferons (IFN-λ1, IFN-α2a, IFN-β, and IFN-γ), and autoantibodies against IFNα and IFNω in the BALF and plasma. The concentrations of cytokines in BALF were ELF-corrected. All numbers are presented as median [IQR] for all groups.

* indicates a difference between the patient group and the healthy volunteers (p<0.05, Dunn’s test).

** indicates a difference between CARDS (+DXM) and non-CARDS (p<0.05, Dunn’s test).

BALF, bronchoalveolar lavage fluid; CARDS, COVID-19 associated acute respiratory distress syndrome; CRP, C-reactive protein; DXM, dexamethasone; IFN-α, interferon α; IFN-α2a, interferon α2a; IFN-β, interferon β; IFN-γ, interferon-γ; IFN-ω, interferon ω; IFN-λ1, interferon λ1; TNF-α, tumor necrosis factor alpha; IL-1β, interleukin 1β; IL-6, interleukin 6; IL-8, interleukin 8; Non-CARDS, non-COVID-19 associated acute respiratory distress syndrome and/or sepsis.

The concentration of TNF-α in the plasma was similar between the patients and healthy controls. In contrast, plasma concentrations of IL-1β, IL-6, and IL-8 were higher in both CARDS and non-CARDS than in healthy controls, and IL-6 values were up to 60-times higher in CARDS −DXM compared to CARDS +DXM (**Table 2**).

### 3.5 Alveolar and systemic levels of IFNs

ELF-corrected alveolar concentrations of IFN-α2a, IFN-β, IFN-γ, and IFN-λ1 were not different between CARDS and non-CARDS patients (**Table 2**). Concentrations of IFN-α2a, IFN-β, and IFN-λ1 in the plasma did not differ between any of the patient subgroups, but IFN-γ was found to be higher in CARDS patients not receiving DXM compared to CARDS patients those receiving DXM and non-CARDS (**Table 2**).

### 3.6 Alveolar and systemic levels of IFN autoantibodies

**Figure 3:**
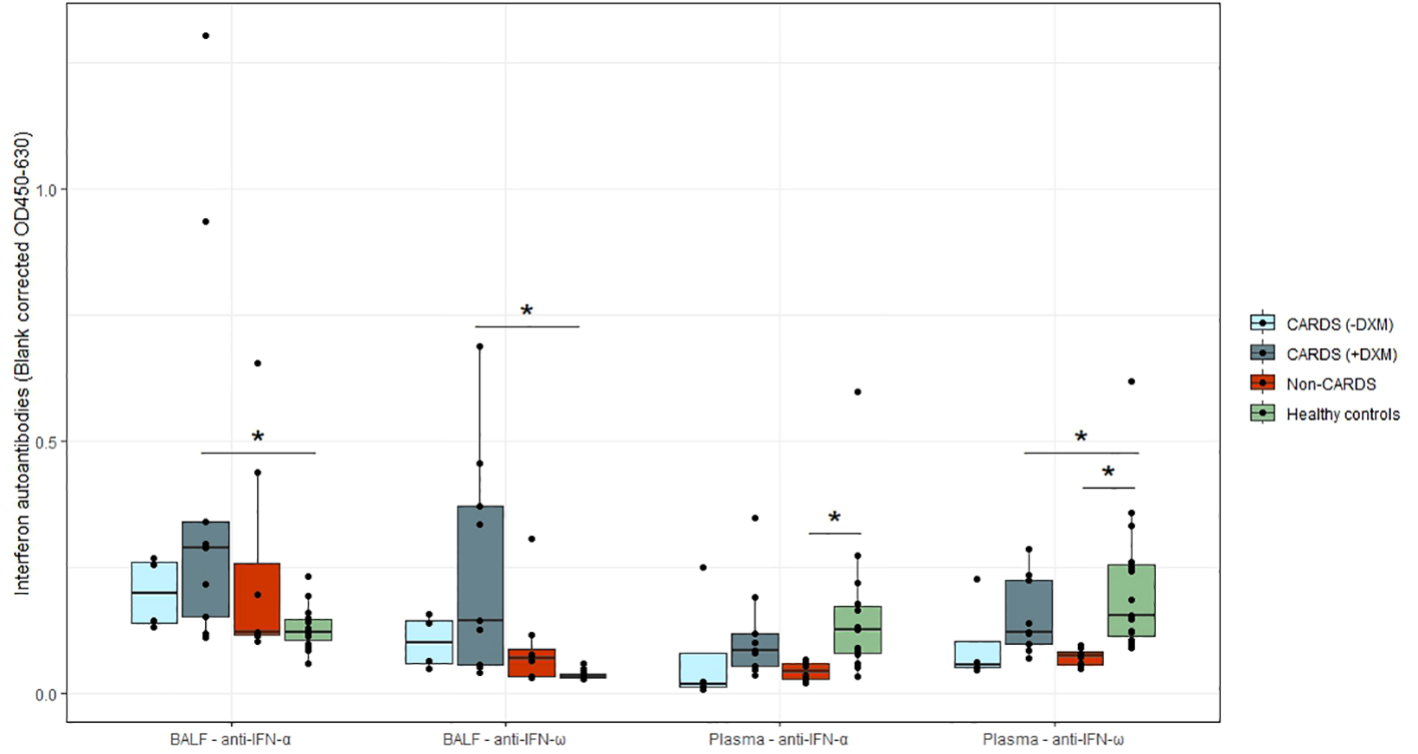
IFNα and IFNω autoantibodies in patients and healthy controls. Levels of alveolar and plasma autoantibodies against IFNα and IFNω with medians (interquartile range, IQR) in CARDS patients without (−DXM, n = 4) or with DXM treatment (+DXM, n = 12), non-CARDS critically ill patients (n = 8) and in healthy controls (n = 15). *P<0.05 (Dunn’s test). Box plots visualize the median, first, and third quartiles (hinges), and whiskers extends from the hinge to the largest value no further than 1.5 ∗ IQR from the hinge, with data beyond plotted individually as outlying points ARDS, acute respiratory distress syndrome; BALF, bronchoalveolar lavage fluid; CARDS, COVID-19 associated acute respiratory distress syndrome; DXM, dexamethasone; IFNα, interferon α; IFNω, interferon ω; Non-CARDS, non-COVID-19 associated acute respiratory distress syndrome and/or sepsis.

ELF-corrected alveolar concentrations of IFNα and IFNω autoantibodies were higher in patients with CARDS than in healthy controls (**Figure 3**). Three patients with CARDS and one patient with non-CARDS had autoantibody levels above 0.5. For both IFNα and IFNω autoantibodies, a difference was found between patients with CARDS receiving DXM and healthy controls (p = 0.042 and p<0.001, respectively).

In the plasma, autoantibody levels of both IFNα and IFNω were found to be significantly lower in healthy volunteers than in non-CARDS patients (p<0.01). Additionally, a difference was observed in IFNω between patients with CARDS not receiving DXM and healthy volunteers (p<0.01). No patients had levels above 0.5 (**Figure 3**).

## 4 Discussion

We evaluated the alveolar milieu of 16 patients with COVID-19-related ARDS and compared it to that of eight critically ill patients with ARDS or sepsis and 15 healthy volunteers by measuring interleukins, interferons, and interferon autoantibodies in both plasma and bronchoalveolar lavage fluid. Patients diagnosed with CARDS presented with evidence of disruption of the alveolo-capillary membrane, whereas both CARDS and non-CARDS patients showed compartmentalization of IL-8. Although IFN autoantibody levels in BALF overall seemed to be low, they were higher in both CARDS and non-CARDS patients than in healthy controls, and 3/16 CARDS patients and 1/8 non-CARDS patients had levels above 0.5. DXM treatment in CARDS seemed to be associated with enhanced alveolo-capillary membrane integrity and lower systemic levels of interleukins and IFN-γ, whereas IL-6 BALF levels seemed to be particularly high in those not receiving DXM.

A hallmark of both CARDS and non-CARDS is increased alveolo-capillary permeability, followed by sequestration of activated neutrophils and accumulation of protein-rich fluid (16–19). In our study, patients with CARDS, particularly the −DXM group, had a higher alveolar protein concentration and a larger degree of gas exchange impairment compared to non-CARDS patients. This is consistent with a recent observational study that found higher index values of pulmonary vascular permeability and extravascular lung water in CARDS vs. non-CARDS patients, which is an estimate of the amount of fluid in the interstitium and alveolar spaces (20). Extravascular lung water may be directly related to the disruption of the alveolo-capillary membrane, possibly caused by severe ongoing systemic and alveolar inflammation, which may be attenuated by the administration of corticosteroids. Accordingly, the RECOVERY trial found that the use of systemically administered DXM markedly reduced mortality in critically ill COVID-19 patients (21), although the mechanisms are largely unknown. The hypothesis that corticosteroids may contribute to preservation or, to some degree, maintain barrier integrity is supported by a previous experimental study in healthy subjects, in which DXM significantly decreased protein leakage compared to placebo after endotoxin-induced lung inflammation (22). Even though it has to be confirmed in larger studies, our results point towards a beneficial effect of DXM in the lungs of patients with CARDS, conceivably due to a more intact alveolo-capillary membrane.

Here, we present absolute, that is ELF-corrected, cytokine concentrations in BALF, which differ from the reported cytokine concentrations in pg/mL of recovered BALF traditionally used in both compartmental and transcompartmental models of experimentally induced inflammation (23) and sepsis (24), and in clinical studies of ARDS (25, 26) and COVID-19 (5). Interestingly, absolute alveolar concentrations of TNF-α, IL-1β, and IL-6 were not different between healthy controls and patients with CARDS and non-CARDS, even though the patient cohort is usually associated with a hyperinflammatory condition. This finding is in contrast with several studies reporting alveolar cytokine concentrations in controls, CARDS, and non-CARDS patients (5, 26, 27), making it imperative to revisit the methodological approach used when comparing alveolar markers of inflammation. In our study, comparable alveolar levels of TNF-α, IL-1β, and IL-6 in healthy controls and patients could be explained by the time factor in CARDS and non-CARDS disease states, with some of these cytokines peaking at a much earlier time point, i.e., before admission to hospital or ICU and thus before collection of samples (28).

Conversely, IL-8 was compartmentalized in the lungs of both CARDS and non-CARDS patients, and IL-8 is a potent chemoattractant, suggesting ongoing alveolar inflammation, which further perpetuates lung injury. Of note, regardless of the methodical approach, the previously mentioned studies did also find IL-8 to be compartmentalized in the lungs (5, 26, 27). Concentrations of IL-6 in BALF and plasma were lower in the CARDS subgroup that received DXM, suggesting a distinct compartmental pathophysiological role of both IL-6 and IL-8. Furthermore, these findings suggest that DXM may attenuate inflammation in CARDS by attenuating the production and/or release of IL-6. In contrast, the alveolar levels of IL-8 were unaffected by the treatment, suggesting that the mechanisms of action in the lungs are not due to the low abundance of IL-8.

Concerning the absolute alveolar concentrations of type I, II, and III IFNs, we found no significant difference between the CARDS and non-CARDS groups. Despite the large number of studies since the beginning of the COVID-19 pandemic investigating the immune response and establishing the crucial role of IFN-I in antiviral defenses against SARS-CoV-2 (15, 29), the exact role of IFNs at the alveolar level remains unknown. However, some studies have found decreased levels of type I IFN (IFN-α2, IFN-β) in both plasma and BALF in the most critical COVID-19 patients compared to patients with only mild-to-moderate COVID-19. Thus, type I IFN may have a critical role, as an impaired response was found to correlate with the degree of severity, emphasizing the importance of type I IFN in antiviral immunity (29, 30).

Several studies investigating autoantibody levels in plasma have shown a correlation between high levels of autoantibodies against type 1 IFNs and mortality in COVID-19 patients (15, 31). To the best of our knowledge, levels of autoantibodies against IFNα and IFNω have only been measured in the BALF of patients with CARDS in another published study (9). They found that 10% of tested patients with life-threatening COVID-19 had neutralizing autoantibodies, which may have contributed to an impaired antiviral type I IFN immune response in the alveolar compartment. In the present study, autoantibodies against IFNα and IFNω were measured in both BALF and plasma, which enhanced our understanding of autoantibody compartmentalization. Assessment of autoantibody levels in the alveolar region is particularly important, as local neutralization of IFNs may lead to a loss of viral control within the lungs. Somewhat surprising, no considerable difference was observed between the two compartments, contrary to what may be expected, with the lungs being the primary site of infection in COVID-19. However, our data showed a tendency towards higher concentrations in BALF, as none of the patients had plasma autoantibody levels in plasma above 0.5 compared with BALF, whereas three CARDS patients had autoantibody levels above the threshold, although the threshold for determining positivity in BAL samples is unknown and it might be incorrect to compare these two. However, when comparing the median values of the concentrations of autoantibodies in both compartments, higher concentrations were observed in BALF. This suggests that autoantibodies may be compartmentalized to some degree.

Corticosteroids are now widely used in the management of community-acquired pneumonia, ARDS, and CARDS (21, 32, 33). These beneficial clinical effects are believed to stem from their ability to modulate the inflammatory response and stabilize the alveolo-capillary membrane (34). While the specific mechanisms underlying these effects remain unknown, recent findings indicate that corticosteroids exert at least some of these immunomodulatory effects through multiple pathways, i.e., by interfering with alveolar B-cell and complement pathway activation (12). The present findings suggest that this also affects IL-6 and IFN-γ signaling.

The present study had several limitations. First, the small sample size introduces the risk of type 2 errors. Thus, alveolar concentrations of interleukins/interferon and autoantibodies and whether corticosteroids have an impact on membrane integrity in CARDS need to be investigated in larger studies. Second, although urea is a widely accepted marker of alveolar fluid dilution, it may still lead to an overestimation of the actual ELF volume, thus limiting the accuracy of ELF solute concentrations. Thus, the reported concentrations of cytokines could be an underestimation of the actual concentrations, which may be even more important to consider in injured lungs. Third, mechanical ventilation *per se* could lead to changes in alveolo-capillary barrier integrity, thus introducing a potential confounding factor without the inclusion of a mechanically ventilated control group. Fourth, it should be noted that during the analysis of cytokines and autoantibodies, only samples from CARDS patients were inactivated by using 0.4% Triton-X. This variation in sample processing may have influenced the results and possibly the comparison between the groups. Lastly, the use of ELF for the calculation of absolute solute concentrations in the alveolar compartment seems appropriate not only to consider as a strength but also a limitation because very few studies in critically ill patients have reported data based on ELF calculations.

Our study had several noteworthy strengths. First, it enrolled all mechanically ventilated patients at an early stage of severe respiratory failure, irrespective of their underlying diseases. This approach enhances the likelihood of accurately depicting the pathophysiological characteristics of both ARDS and CARDS. Second, the BAL procedure was performed according to the protocol by the same clinician across both patient cohorts and healthy volunteers, thus mitigating the potential for sample variability by ensuring the consistent use of identical lung segments and flush volumes. Third, our investigation included COVID-19 patients treated with and without DXM, thereby providing additional support for the hypothesis that dexamethasone may impact the integrity of the alveolo-capillary membrane.

In conclusion, while both CARDS and non-CARDS displayed compartmentalization of IL-8, regardless of the disease entity, patients with CARDS specifically showed evidence of increased alveolo-capillary membrane permeability. This phenomenon appears to be associated with numerous alveolar and systemic immune pathways, including a pronounced increase in alveolar IL-6 levels. This may potentially be modulated by DXM treatment, providing a mechanistic link to its effect on alveolo-capillary membrane integrity, which could contribute to the more favorable outcome of patients with CARDS receiving corticosteroids.

## Data availability statement

The raw data supporting the conclusions of this article will be made available by the authors, without undue reservation.

## Ethics statement

The studies involving humans were approved by Ethical Committee of the Capital Region of Copenhagen (H-2-2009-131; H-2-2011-021; H-2-0023-159). The studies were conducted in accordance with the local legislation and institutional requirements. Written informed consent for participation in this study was provided by the participants’ legal guardians/next of kin.

## Author contributions

TJ: Writing – original draft. SJ: Writing – review & editing. NM: Writing – review & editing. TM: Writing – review & editing. RB: Writing – review & editing. AR: Writing – review & editing. RP: Writing – review & editing.
